# Utilisation of a GP-staffed emergency response unit: an observational study from Norway

**DOI:** 10.3399/BJGPO.2025.0101

**Published:** 2026-01-28

**Authors:** Anders Rønning, Ann-Chatrin Linqvist Leonardsen, Odd Martin Vallersnes, Magnus Hjortdahl

**Affiliations:** 1 Fredrikstad and Hvaler Emergency Primary Care Centre, Fredrikstad, Norway; 2 Østfold University College, Halden, Ostfold, Norway; 3 Department of Surgery, Østfold Hospital Trust, Sarpsborg, Norway; 4 Department of General Practice, University of Oslo, Oslo, Norway; 5 Oslo Accident and Emergency Outpatient Clinic, Oslo, Norway; 6 Oslo Metropolitan University, Oslo, Norway; 7 University of Tromsø, Tromsø, Norway

**Keywords:** trauma, general practitioners, primary healthcare, emergency treatment

## Abstract

**Background:**

Emergency department (ED) crowding is a growing challenge, highlighting the need for safe and effective prehospital alternatives to hospital conveyance.

**Aim:**

To investigate how a GP-staffed emergency primary care response unit (EPCRU) affects resource allocation and patient pathways.

**Design & setting:**

A prospective observational study conducted in two Norwegian municipalities served by a GP-staffed EPCRU.

**Method:**

All call-outs (*n* = 2950) performed by the EPCRU from 1 April 2023 to 31 March 2024 were included. Data on dispatch origin, triage (telephone and on site), reason for dispatch, level of care, and participating services were analysed.

**Results:**

Most call-outs were categorised as acute (57.6%) by the call centre and comprised a broad spectrum of conditions and severities. The EPCRU was first on scene in 44.1% of cases. In total, 44.1% of patients were treated on site without hospital conveyance. A mismatch was observed between telephone and on-site triage: 34.2% of acute cases were reassigned to yellow (the midpoint of a 5-point urgency scale) by the Rapid Emergency Triage and Treatment System (RETTS) on-site triage. The EPCRU altered expected care trajectories compared with standard ambulance response by enabling both non-conveyance and direct ED admissions.

**Conclusion:**

A GP-staffed response unit may enhance resource efficiency, patient flow, and timely care, which offers potential benefits for emergency systems facing growing demand.

## How this fits in

Healthcare systems are under increasing capacity pressure, with acute medical contacts representing a considerable contributor. Several European countries involve GPs in prehospital emergency care, but evidence on their role remains limited. This observational study describes a Norwegian GP-staffed emergency primary care response unit, finding that more than half of patients were managed without hospital admission. The results suggest that GP involvement can support more appropriate patient pathways, reduce unnecessary hospital conveyance, and strengthen emergency care capacity.

## Introduction

Increasing patient inflow to emergency departments (EDs) and rising hospital admission rates contribute to mounting capacity challenges in healthcare systems.^1,2^ This strain is expected to intensify further owing to a growing proportion of older individuals living at home with chronic conditions, combined with ongoing shortages in healthcare personnel.^3^ Prehospital emergency medicine services (EMS) will often be the first to assess patients, and therefore play an important role in patient pathways, ensuring that patients are triaged to the right level of care at the right time.^4^

Crowding in EDs is a well-documented risk factor for adverse events, highlighting the importance of safe alternatives to hospital conveyance.^5^ A Finnish cohort study found that four in five of nearly 12 000 non-conveyed patients did not have any re-contact in the follow-up period, suggesting that EMS non-conveyance is a safe method of avoiding ED crowding.^6^ However, without specific training in urgent or primary care assessments, ambulance personnel may struggle with conveyance decisions. This can result in unnecessary ED transfers or missed opportunities for senior clinical input.^7^ A Norwegian study found that patients were twice as likely to be transported directly to the hospital when the on-call GP was not notified of the call-out.^8^ An English study found that patients assessed by a GP via telephone, rather than face to face, had a higher likelihood of being transported to the hospital.^9^ When ED conveyance is unnecessary, patients often still require urgent management. Involvement of GPs in EMS has been proposed as a measure to improve patient care and resource utilisation.^10,11^ A 2021 study found that 17 of the 32 surveyed European countries had a system involving GPs in prehospital EMS.^12^

The specific contribution of GPs, particularly in settings with dedicated emergency response units, remains underexplored.^7^ Consequently, the aim of this study was to investigate the impact of a GP-staffed emergency primary care response unit (EPCRU) on resource allocation and patient pathways.

## Method

### Design

The study had a prospective, observational design, investigating EMS call-outs conducted by an EPCRU in two Norwegian municipalities over a 1-year period.

### Norwegian emergency health care

**Figure 1.**
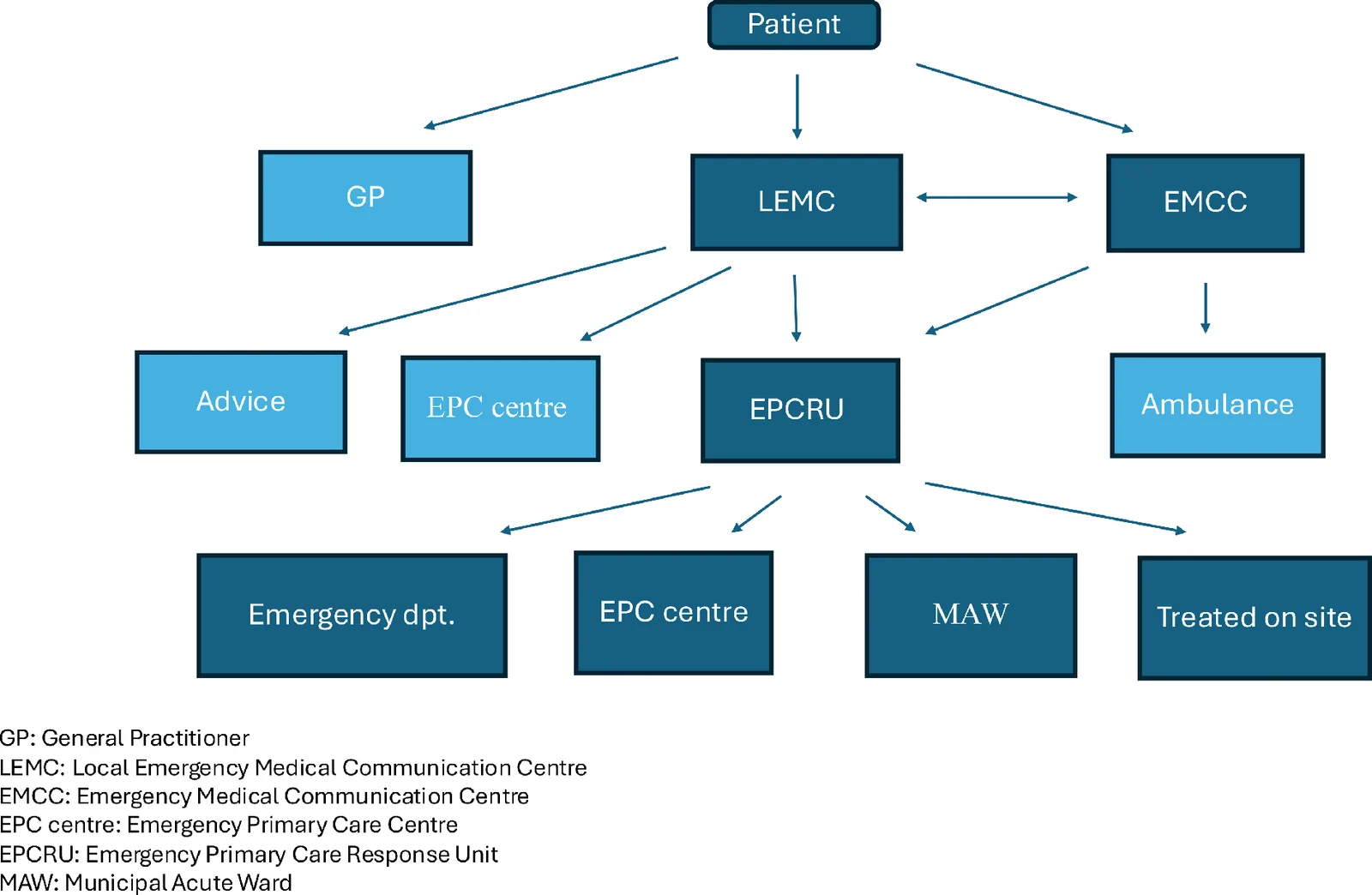
The role of the EPCRU in patient pathways. EMCC = emergency medical communication centres. EPC = emergency primary care. EPCRU = emergency primary care response unit. LEMC = local emergency medical communication centre. MAW = municipal acute ward.

Norway has a two-level EMS system, with primary care managed by the municipalities and secondary care managed by the state.^13,14^ The emergency medical communication centres (EMCCs), part of secondary care, are call centres for time-critical medical emergencies. The EMCC dispatcher will evaluate the call and allocate adequate resources. For non-life-threatening emergencies, patients contact their GP during working hours. If unavailable, the local emergency medical communication centre (LEMC), part of primary care, can be contacted. A nurse at the LEMC will triage the call and take appropriate action, such as providing advice, directing the patient to the GP-staffed local emergency primary care (EPC) centre, or notifying the on-call GP (hereafter referred to as the EPC physician), who will determine whether on-site assessment is required (Figure 1).

Both EMCCs and many LEMCs use the Norwegian Index for Emergency Medical Assistance as a tool for telephone triage.^15^ In a suspected life-threatening condition, the EMCC will dispatch ambulance resources and alert the local EPC physician, who may respond at their discretion. EPC centres are obliged to ensure their personnel are trained and equipped for emergencies, including performing diagnostics and providing interventions.^13^ The degree of training and equipment available vary across municipalities. A 2024 survey by the National Centre for Emergency Primary Health Care found that 86 out of 167 EPC centres (51%) had an EPCRU.^16^

### Setting

The study was conducted at an EPC centre responsible for providing 24/7 urgent care across two municipalities, Fredrikstad and Hvaler, which cover an area of 383 km^2^, encompassing coastal islands, forests, and urban areas. The two municipalities have 90 345 registered residents (as of 1 July 2024), with a considerable increase in population during the summer months. The longest drivable distance to the nearest hospital is 55 km, in addition to islands where transport must be arranged by boat. There are typically three or four ambulances in operation, in addition to a search-and-rescue vessel, at any given time in the area. Additionally, helicopter emergency services with an anaesthesiologist can be dispatched, if available.

In October 2022, the EPC centre introduced an EPCRU, staffed by a selected and trained team of EPC nurses and EPC physicians with significant experience in general practice. The team is equipped to manage a wide spectrum of acute and subacute medical conditions, including access to point-of-care laboratory tests and ultrasound equipment. The EPCRU primarily operates from 8.00 am to 10.00 pm on weekdays and from 11.00 am to 9.00 pm on weekends and holidays. Whether the EPCRU is dispatched is based on the individual judgement of the EPC physician on call.

### Sample

All call-outs (*n* = 2950) carried out by the EPCRU, during the period from 1 April 2023 to 31 March 2024, were included.

### Data collection

Information about all call-outs was recorded on a paper form by the EPCRU nurse. Call-out identification was recorded using the patient’s initials and date of birth, which were cross-referenced with the EPC centre medical records.

‘Dispatcher’ referred to the entity responsible for initiating a dispatch; EMCC or LEMC.

Telephone triage was categorised and recorded based on the dispatcher’s assessment of the patient’s condition, according to the Norwegian Index for Medical Emergency Assistance before dispatch.^15^ Calls are triaged into three categories: acute, indicating the need for immediate response; urgent, indicating intermediate urgency; and non-urgent, indicating low degree of urgency.

Reason for dispatch was documented as the reason for initiating the call-out, as assessed by the EPCRU physician. These reasons were subsequently categorised by the research team into 16 groups Table 1. The 16 groups are not identical with the categories in Norwegian Index for Medical Emergency Assistance.

**Table 1.** Characteristics of EPCRU call-out (*n* = 2950)

	N (%)
**Dispatcher (** ***n*** **= 2950)**	
LEMC	1038 (35.2)
EMCC	1905 (64.6)
Missing	7 (0.2)
**Telephone triage (** ***n*** **= 2950)**	
Acute	1698 (57.6)
Urgent	1125 (38.1)
Non-urgent	124 (4.2)
Missing	3 (0.1)
**Reason for dispatch (** ***n*** **= 2950)**	
Respiratory	350 (11.8)
Trauma	301 (10.2)
Impaired consciousness	299 (10,1)
Infection	271 (9.2)
Chest pain	235 (8.0)
Psychiatric disease	223 (7.6)
Central nervous system symptoms	219 (7.4)
Musculoskeletal disorders	105 (3.6)
Cardiac arrest	95 (3.2)
Seizures	83 (2.8)
Death	83 (2.8)
Loss of function	81 (2.7)
Abdominal pain	79 (2.7)
Confusion	78 (2.6)
Violence	24 (0.8)
Other	423 (14.3)
Missing	1 (0.2)
**First unit on scene, telephone triage acute call-outs (** ***n*** **= 1698)**	
Ambulance	780 (45.9)
EPCRU	749 (44.1)
EPCRU /ambulance simultaneously	127 (7.5)
Fire department	21 (1.2)
Missing	21 (1.2)
**RETTS (** ***n*** **= 2950)**	
Red	304 (10.3)
Orange	636 (21.6)
Yellow	1070 (36.3)
Green	539 (18.3)
Blue	165 (5.6)
Missing	236 (8.0)
**Level of care (** ***n*** **= 2950)**	
Hospital	1426 (48.3)
Treated on site	1300 (44.1)
Municipal acute ward	114 (3.9)
EPC	90 (3.1)
Missing	20 (0.7)
**Participating units (** ***n*** **= 2950)**	
Ambulance	1784 (60.5)
Police	339 (11.5)
Fire department	154 (5.2)

EMCC = emergency medical communication centre. EPCRU = emergency primary care response unit. LEMC = local emergency medical communication centre. RETTS = Rapid Emergency Triage and Treatment System.

‘First unit on scene’ referred to the first emergency service that arrived at the location of the incident when triaged as ‘acute’ by the dispatch centre (EPCRU, ambulance, fire department, EPCRU and ambulance arriving together).

The Rapid Emergency Triage and Treatment System (RETTS) is a triage system for assessing the severity of a patient’s condition. It categorises urgency into the following five levels: red (most urgent); orange; yellow; green; and blue (least urgent). The system combines vital signs, reported symptoms, and mechanism of injury. The most critical finding — regardless of whether it stems from physiological parameters, presenting symptoms, or trauma mechanism — determines the final triage level.^17^ The triage was conducted by the EPCRU nurse on arrival, with the EPCRU physician blinded to the results. RETTS is also used by the local ambulance service to decide the clinical pathway when they see a patient by themselves. Patients triaged red and orange are transported directly to the hospital. Patients triaged yellow, green, and blue are transported to their regular GP or the EPC centre. If emergency medical technicians assess that the patient can likely remain on site without further medical treatment, they are, according to local guidelines, required to consult a physician.

Level of care denoted the treatment facility to which the patient was transported for further medical care (hospital, EPC centre, municipal acute ward [MAW], or treated on site). MAW is a short-term facility within the primary healthcare system, designed for patients who require observation or treatment, but not hospital-level care.

Participating units listed the collaborating emergency services involved in each call-out (ambulance, police, or fire department).

### Analysis

Descriptive data of EPCRU call-outs were presented using absolute numbers and percentages. Analyses were done using the Statistical Package for the Social Sciences (SPSS; version 29).

## Results

### Characteristics of EPCRU call-outs

The EPCRU was dispatched to a wide range of call-outs, involving patients with diverse clinical presentations. The severity ranged from cases requiring life-saving interventions to conditions that were ultimately benign. Most missions were categorised as acute (*n* = 1698, 57.6%), and dispatched from the EMCC (*n* = 1905, 64.6%) (Table 1). The most frequent reason for dispatch was respiratory distress (*n* = 350, 11.8%), followed by trauma (*n* = 301, 10.2%), and impaired consciousness (*n* = 299, 10.1%). The first unit on scene was evenly distributed; ambulance (*n* = 780, 45.9%), and EPCRU (*n* = 749, 44.1%), or simultaneously (*n* = 127, 7.5%). The most frequent RETTS score was yellow (*n* = 1070, 36.3%). Level of care was most commonly hospital (*n* = 1426, 48.3%), followed by treated on site (*n* = 1300, 44.1%). The ambulance service was the most frequent co-responder (*n* = 1784, 60.5%).

### Telephone triage versus in person triage

**Figure 2.**
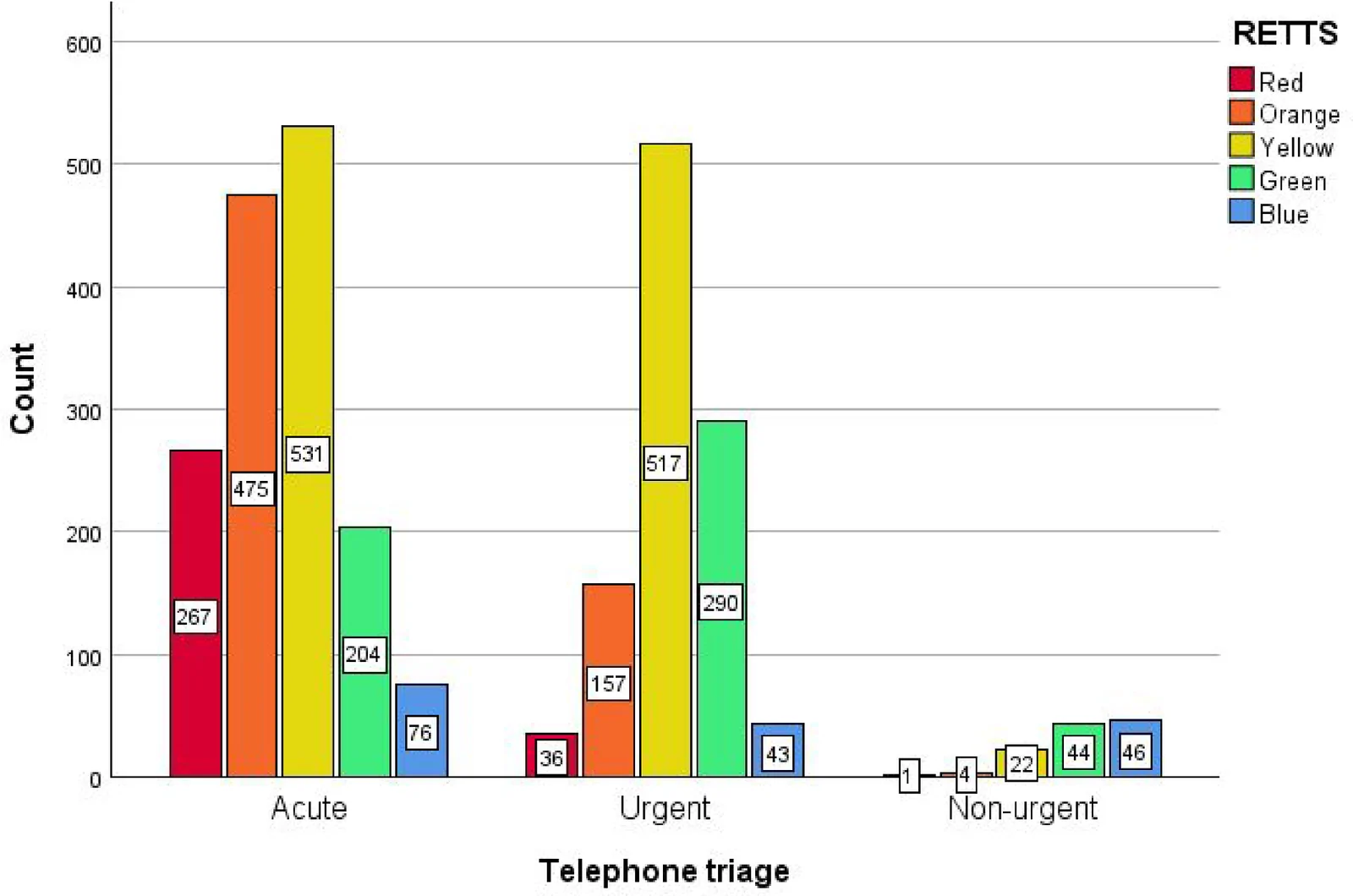
Telephone triage versus in person triage (Rapid Emergency Triage and Treatment System; RETTS)

The urgency level assigned by the dispatch centre did not always correspond with the patient’s condition as assessed on site by the EPCRU (Figure 2). A substantial proportion of patients initially categorised as acute were later deemed to be in a less critical state by on-site RETTS assessment: 531 (34.2%) were reassigned to yellow, and 475 (30.6%) were categorised as orange. However, some patients were assigned a higher urgency level following on-site RETTS assessment by the EPCRU nurse. Among those initially categorised as urgent by the dispatch centre, 36 (3.5%) were reclassified as red, and 157 (15.1%) as orange.

### Level of care assessed by telephone triage

**Figure 3.**
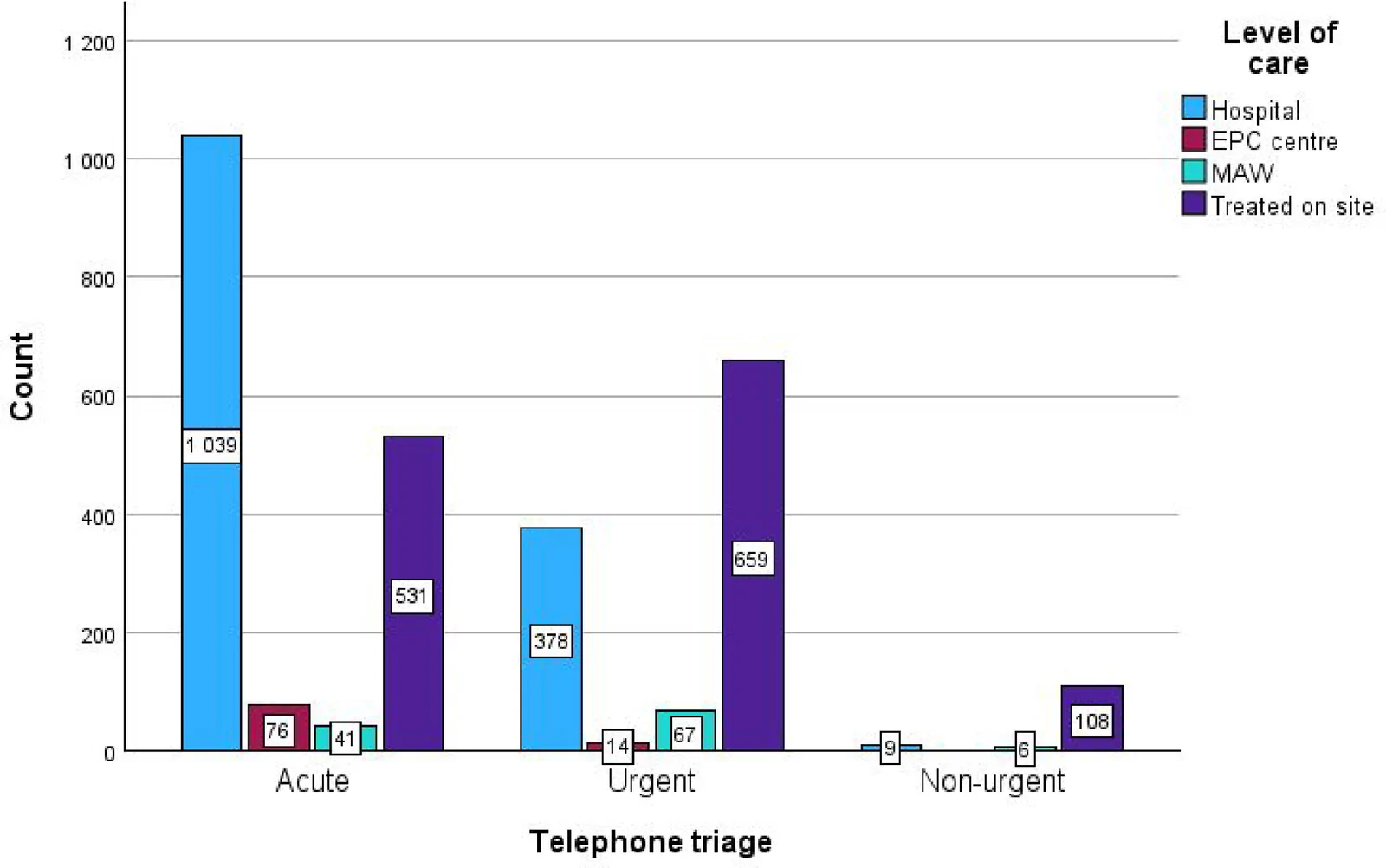
Telephone triage and level of care. EPC = emergency primary care. MAW = municipal acute ward

Figure 3 shows the correlation between telephone triage urgency levels and the level of care provided. The initial urgency assessment did not always align with level of care needed. Among patients classified as acute, 1039 (61.6%) were hospitalised, while 531 (31.5%) were treated on site. For those triaged as urgent, 659 (58.9%) were treated on site, but a substantial portion of the patients were also admitted to the hospital, 378 (33.8%).

### Different level of care when the EPCRU was involved

**Figure 4.**
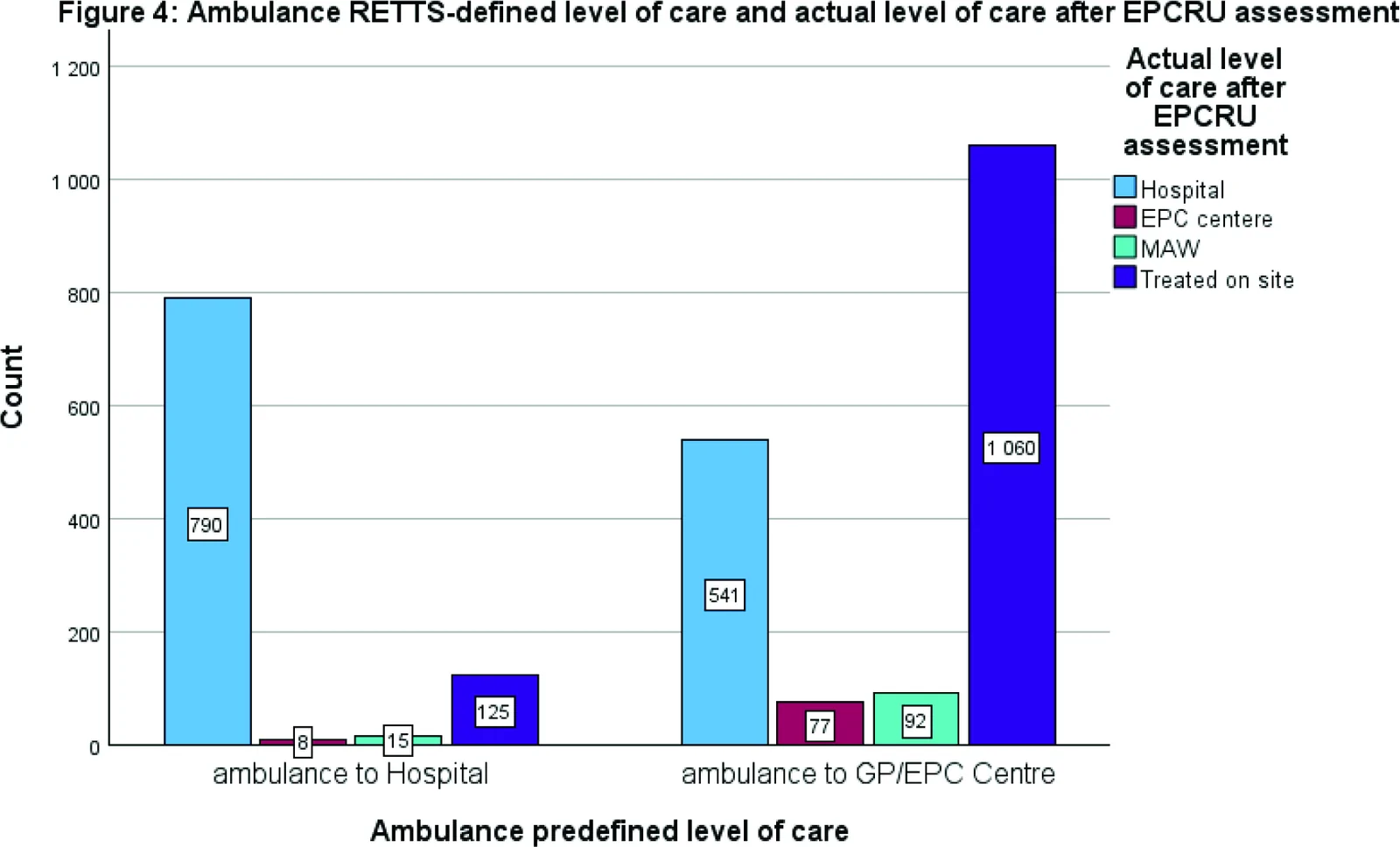
Ambulance RETTS-defined level of care and actual level of care after EPCRU assessment. EPC = emergency primary care. EPCRU = emergency primary care response unit. MAW = municipal acute ward. RETTS = Rapid Emergency Triage and Treatment System

The involvement of the EPCRU appears to influence patient pathways, resulting in deviations from standard transport recommendations based on RETTS. Figure 4 shows that 148 (15.8%) of patients triaged as RETTS red or orange, whom the ambulance would have taken directly to the hospital, received lower levels of care. Among patients that according to the RETTS system would have been transported by ambulance to their regular GP or the EPC centre, 541 (30.6%) were transported directly to hospital, and 1060 (59.9%) were treated on site. Overall, 51.1% of patients (*n* = 1504) were managed without hospital admission.

## Discussion

### Summary

EPCRU call-outs were mostly initiated by the EMCC and comprised a broad spectrum of conditions and severities. In 44.1% of the cases, the EPCRU was the first emergency service to arrive on site. When the EPCRU was dispatched, 1504 (51.1%) of the patients received treatment without requiring transport to hospital (treated on site, MAW, and EPC centre). Triage from the dispatch centres did not consistently align with triage on site or the subsequent level of care. Participation of the EPCRU impacted the patient pathway by facilitating direct hospital admission for a considerable portion of patients who, according to RETTS, would otherwise have been initially transported to their GP or the EPC centre, and by providing on-site treatment for others who would have been transported to higher levels of care.

### Strengths and limitations

A key strength of this study is the availability of a complete dataset covering all call-outs for a GP-staffed EPCRU, including the level of care the patient ultimately received. Another strength is the inclusion of RETTS scores, which provide an objective assessment of the patient’s condition and offer insight into what the outcome might have been if the patient had only been assessed by an ambulance crew.

As a descriptive observational study without a control group, the findings should be interpreted with caution. The single-centre design limits generalisability, and the absence of patient outcomes, such as discharge diagnoses and mortality, restricts conclusions about care quality and safety.

### Comparison with existing literature

The wide range of medical conditions and varying degrees of severity encountered by the EPCRU team align with findings from the London Physician Response Unit, where the involvement of generalist emergency physicians supported effective, patient-centred care across a broad spectrum of emergencies.^18^ That study also highlights the value of interagency collaboration with community healthcare providers in delivering coordinated emergency care. Similarly, the high proportion of call-outs involving interagency collaboration in our data emphasises the need for joint training and a shared understanding of roles across emergency services to enable seamless patient management.

A Norwegian study from 2019 exploring the peculiarities of working at the EMCC described the inherent difficulties in determining appropriate responses during emergency calls.^19^ This aligns with our finding of a low correlation between initial urgency assessments and actual severity of the patient’s condition. These uncertainties add to the complexity of the decision making when assessing whether to participate in an EMS call-out, as described by GPs working in rural Norway.^11^

Overtriage at the dispatch level likely contributed to the high proportion of urgent calls in which patients were treated on site. This was also found in a 2023 Finnish study, which identified substantial challenges related to overtriage in dispatch centre assessments.^20^ While overtriage can help ensure rapid assistance in uncertain cases, it also poses a risk for responding personnel and their surroundings by frequent high-speed response. Moreover, a high degree of overtriage may strain resources in an already limited healthcare system.^21^

The EPCRU responded to several critically ill patients, including those with cardiac arrest, trauma, and suspected sepsis. Studies examining prehospital physician involvement in such conditions suggest that the presence of a physician is associated with improved patient outcomes.^22–24^ While treatment outcomes were not directly assessed, the prevalence of critically ill patients highlights the potential relevance of physician-led prehospital care for this population. Beyond providing physician-led care in cases of critical illness, participation in acute call-outs also enables the EPCRU to initiate non-conveyance when appropriate, as one-third of these patients were treated on site. The large proportion of acute call-outs in which the EPCRU was the first emergency service on scene underlines its role in strengthening overall emergency preparedness.

In our cohort non-conveyance was initiated in 44.1% of call-outs. These findings are consistent with those reported by an Irish response unit — staffed by an emergency physician and an emergency medical technician attending low-acuity calls — which recorded a non-conveyance rate of 68%.^25^ Furthermore, a 2023 systematic mapping review suggested that EMS models involving GPs or physicians may reduce the likelihood of emergency department conveyance and hospital admission in selected patient groups.^7^ In comparison, the preliminary results of an unpublished 2021 Norwegian study reported a non-conveyance rate of 7.3% in conventional EMS services.^26^ In contrast, a ‘single responder’ paramedic model in a Danish context demonstrated a non-conveyance rate of 29.1%, highlighting how non-transport decisions vary considerably depending on provider role and system design.^27^

### Implications for research and practice

The integration of a GP-staffed EPCRU into the EMS may facilitate alternative care pathways outside of traditional ED visits.

Future research, including use of control groups (for example, standard ambulance response without GP involvement), should explore the impact of prehospital diagnostics performed by GPs on patient pathways and outcomes. To further evaluate the safety of EPCRU-care, a follow-up study is underway that will examine hospital readmissions and mortality among patients who were treated on site or diverted from hospital admission. Further studies should also examine the relationship between urgency assessments made by call centres and actual patient urgency.

In conclusion, the integration of a GP-staffed response unit into prehospital emergency care demonstrates potential in optimising resource allocation, improving patient pathways, reducing unnecessary hospital admissions, and ensuring timely physician-led interventions, underscoring the value of GP involvement in emergency healthcare systems facing increasing capacity challenges.

## Data

The dataset is not publicly available.
